# Supplementary material for: Quasiperiodic disorder induced critical phases in a periodically driven dimerized p-wave Kitaev chain
Source: Sci Rep. 2024 Sep 4;14:20603. doi: 10.1038/s41598-024-70995-2 (PMC11375019; doi:10.1038/s41598-024-70995-2)
Supplement: Supplementary file 1 — Supplementary Information. [file 41598_2024_70995_MOESM1_ESM.pdf]

Supplementary material for Quasiperiodic disorder induced critical phases in a periodically driven dimerized p-wave Kitaev chain

Koustav Roy[1], Shilpi Roy[1,2], and Saurabh Basu[1]

[1]*Department of Physics, Indian Institute of Technology Guwahati-Guwahati, 781039 Assam, India*

[2]*Department of Physics, National University of Singapore, 117542, Singapore*

I. WINDING NUMBER FOR A UNIFORM DRIVE

In addition to an incommensurate quasiperiodic (QP) potential, one can also evaluate bulk invariants corresponding to a homogeneous drive, that is $\mu_A = \mu_B = \lambda \sum_{m=-\infty}^{m=\infty} (t - mT)$. Under periodic boundary conditions, we can write down the static counterpart of the Hamiltonian in momentum space as,

$$H = \begin{pmatrix} -\mu & P(k) & 0 & Q(k) \\ P^*(k) & -\mu & -Q^*(k) & 0 \\ 0 & -Q(k) & \mu & -P(k) \\ Q^*(k) & 0 & -P^*(k) & \mu \end{pmatrix}, \tag{1}$$

where,

$$P(k) = -t[(1 + \delta) + (1 - \delta)e^{-ika}] \tag{2}$$

and

$$Q(k) = \Delta[(1 + \delta) - (1 - \delta)e^{-ika}] \tag{3}$$

Note that the effective Hamiltonian, as in Eq. 7 of the main text, for a uniform drive as above does not necessarily pick up the same symmetry as that of the original Hamiltonian. Moreover, we are interested in a $\mu \neq 0$ situation, where the chiral symmetry is defined by, $\hat{C} = \hat{\sigma}_x \otimes \hat{\sigma}_0$, such that the Hamiltonian belongs to the BDI class. Hence, the computation of bulk invariants under the same classification corresponding to a driven scenario requires a pair of symmetric time frames. Following the expressions given in Eq. 9 and Eq. 10, one can obtain the effective Hamiltonian, H_{eff}^m $(m = 1, 2)$ corresponding to the symmetric time frames [1–3]. Hence, the topological phases of the system can be characterized by a pair of winding numbers that satisfy Eq. 14 of the main text.

Further, the evaluation of the winding numbers in each frame requires an introduction of a unitary operator ($\hat{U}_s$) constructed using the chiral basis, with the help of which, H_{eff}^m can be made off-diagonal in its canonical (chiral) basis representation. For a $\mu \neq 0$ scenario, the unitary operator, $\hat{U}_s$ takes the form,

$$\hat{U}_s = \frac{1}{\sqrt{2}} \begin{pmatrix} \hat{I} & \hat{I} \\ -i\hat{I} & i\hat{I} \end{pmatrix}, \tag{4}$$

such that, if $\hat{U}_s \hat{C} \hat{U}_s^\dagger = \text{diag}(\hat{I}, -\hat{I})$, then,

$$\hat{U}_s \hat{H}_{\text{eff}}^m \hat{U}_s^\dagger = \begin{pmatrix} 0 & S(k) \\ S^\dagger(k) & 0 \end{pmatrix} \tag{5}$$

where,

$$S(k) = \begin{pmatrix} -i\mu & i(P(k) - Q(k)) \\ i(P^*(k) + Q^*(k) & -i\mu \end{pmatrix} \tag{6}$$

Now, one can define the chiral index by the following expression,

$$\nu^m = \frac{1}{2\pi i} \int_{-\pi/a}^{\pi/a} dk \partial_k \ln S(k) \tag{7}$$

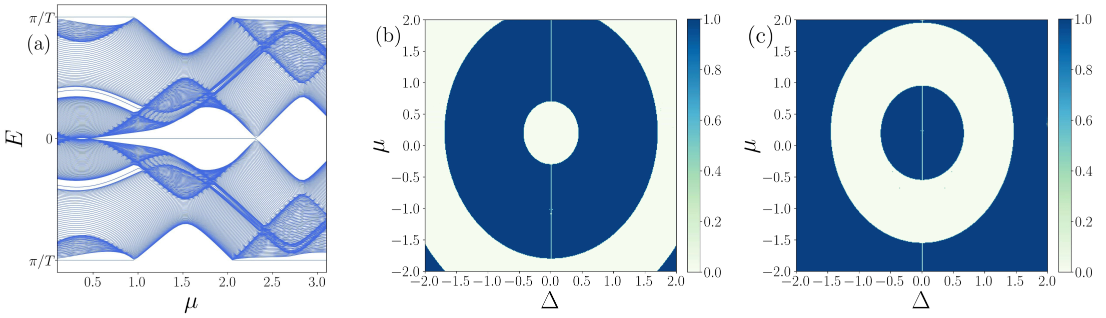

FIG. 1: (a) shows the Floquet quasi-energy spectrum inside the first FBZ, as a function of μ and with fixed Δ, say $\Delta = 0.5$. The topological phase transitions are marked by the gap closing at $E = 0$ and π/T with the corresponding appearance or disappearance of the zero and π energy modes. (b)-(c) depict the topological phase diagram in the $\mu - \Delta$ plane, computed using the winding numbers corresponding to the zero (ν^0) and the π (ν^π) energy modes, respectively. The parameters used are, $\delta = 0.6, \lambda = 0.5$.

One can evaluate the pair of winding numbers (ν^0, ν^π) by following Eq. 14. Fig. 1 shows topological phase diagram in terms of ν^0 and ν^π respectively, plotted in $\mu - \Delta$ plane. The results correctly predict the number of zero and π edge modes corresponding to the real space spectrum shown in Fig. 1(a). Additionally, a line along $\Delta = 0$ signifies the importance of particle-hole symmetry in protecting the topology of the system and is shown in Fig. 1(b) and Fig. 1(c).

II. METHOD FOR CALCULATIONS

Our research focuses on the properties of the dimerized Kitaev chain model, where the Hamiltonian is written as an $N \times N$ matrix (Eq. 1 of the main text) in the site basis. To incorporate the effects of the periodic driving of the system, the Hamiltonian is allowed to evolve over a time period using the time-ordered product of the exponential matrices to obtain the Floquet effective Hamiltonian, as shown in Eq. 4 of the main text, which again appears in the form of an $N \times N$ matrix. Hence, a diagonalization method is used to compute the eigenvalues and eigenvectors of the matrices (both Eq. 1 and 4). These eigenvalues were plotted as a function of various parameters of the Hamiltonian to obtain the energy spectrum and identify the zero Majorana modes, as illustrated in Fig. 5. Further, the eigenvalues were used in our analyses, for the computation of the level spacing statistics (Eq. 24) and the Hausdorff dimension (Eq. 26). Also, the eigenvectors were employed to compute the real-space winding number (Eq. 28) and also the participation ratios (Eq. 19 and 20) which aid us in studying the localization properties, as shown in Fig. 9. Further, the fractal dimension (Eq. 23) too has been obtained using these eigenvectors and demonstrated in Fig. 11.

[1] K. Roy and S. Basu, Topological properties of a periodically driven Creutz ladder, Phys. Rev. B **108**, 045415. https://doi.org/10.1103/PhysRevB.108.045415 (2023).
[2] L. Zhou and J. Gong, Floquet topological phases in a spin-1/2 double kicked rotor, Phys. Rev. A **97**, 063603. https://doi.org/10.1103/PhysRevA.97.063603 (2018).
[3] J. K. Asbóth, B. Tarasinski, and P. Delplace, Chiral symmetry and bulk-boundary correspondence in periodically driven one-dimensional systems, Phys. Rev. B **90**, 125143. https://doi.org/10.1103/PhysRevB.90.125143 (2014).
